# CsMAP34, a teleost MAP with dual role: A promoter of MASP-assisted complement activation and a regulator of immune cell activity

**DOI:** 10.1038/srep39287

**Published:** 2016-12-23

**Authors:** Mo-fei Li, Jun Li, Li Sun

**Affiliations:** 1 Key Laboratory of Experimental Marine Biology, Institute of Oceanology, Chinese Academy of Sciences, Qingdao, China; 2 Laboratory for Marine Biology and Biotechnology, Qingdao National Laboratory for Marine Science and Technology, Qingdao, China; 3 School of Biological Sciences, Lake Superior State University, Sault Ste Marie, MI, USA

## Abstract

In teleost fish, the immune functions of mannan-binding lectin (MBL) associated protein (MAP) and MBL associated serine protease (MASP) are scarcely investigated. In the present study, we examined the biological properties both MAP (CsMAP34) and MASP (CsMASP1) molecules from tongue sole (*Cynoglossus semilaevis*). We found that CsMAP34 and CsMASP1 expressions occurred in nine different tissues and were upregulated by bacterial challenge. CsMAP34 protein was detected in blood, especially during bacterial infection. Recombinant CsMAP34 (rCsMAP34) bound *C. semilaevis* MBL (rCsBML) when the latter was activated by bacteria, while recombinant CsMASP1 (rCsMASP1) bound activated rCsBML only in the presence of rCsMAP34. rCsMAP34 stimulated the hemolytic and bactericidal activities of serum complement, whereas anti-CsMAP34 antibody blocked complement activities. Knockdown of CsMASP1 in *C. semilaevis* resulted in significant inhibition of complement activities. Furthermore, rCsMAP34 interacted directly with peripheral blood leukocytes (PBL) and enhanced the respiratory burst, acid phosphatase activity, chemotactic activity, and gene expression of PBL. These results indicate for the first time that a teleost MAP acts one hand as a regulator that promotes the lectin pathway of complement activation via its ability to recruit MBL to MASP, and other hand as a modulator of immune cell activity.

The complement system is activated via three major pathways, of which the lectin pathway serves as the first line of defense against microbial intruders^1^. It is activated when mannan-binding lectin (MBL) or ficolins binds appropriate carbohydrate or acetylated patterns of microbes^2^. Binding of MBL to a target leads to activation of mannan-binding lectin associated serine proteases (MASPs), which then cleave complement factors C4 and C2, resulting in the formation of the C3 convertase, C4b2a^3^,^4^. The C3 convertase is able to cleave the central complement component C3 into C3a and C3b^5^. C3b binds the C3 convertase to form C5 convertase, which cleaves C5 to C5a, a potent anaphylatoxin, and C5b^6^. C5b recruits C6, C7, C8, and C9 molecules to assemble the terminal membrane attack complex (MAC)^1^, which creates a hole or pore in the membrane that can kill or damage the pathogen or cell^7^.

In humans, three serine proteases have been reported and named MASP1, MASP2, and MASP3^2^. In addition, two nonenzymatic MASPs have also been found and named mannan-binding lectin associated protein (MAP) 44 and MAP19^8^,^9^,^10^. MASP1, MASP3, and MAP44 are the alternative splice products of the MASP1/3 gene, and MASP2 and MAP19 are encoded by the MASP2 gene^8^,^9^,^11^. MASPs contain five regulatory domains (CUB-EGF-CUB-CCP-CCP) and a serine protease domain. The regulatory domains of MASP1 and MASP3 are consistent, as they are derived from the same gene, but their serine protease domains are different^11^. MAP44 contains the first four domains of MASP1 and an additional short peptide^8^,^9^. MAP19 only contains two regulatory domains (CUB-EGF)^12^. The domains of CUB-EGF-CUB are involved in Ca^2+^ dependent association with the recognition molecules^13^,^14^.

MASP2 is necessary for the operation of the lectin pathway^3^. MASP2 can autoactivate and subsequently cleave C4 and C2, leading to the formation of C4b2a^15^,^16^. Some recent studies indicate that MASP2 can also be activated by MASP1 in complex with MBL, which is responsible for 60% of the C2 cleavage^17^,^18^,^19^. Therefore, both MASP1 and MASP2 may be essential for the lectin pathway of complement activation^20^. MASP3 is recently shown to be activated by MASP1, and it may be involved in the activation of the alternative pathway^21^. The exact roles of MAP19 and MAP44 remain to be clarified^22^. To date, the precise function and mechanism of MASPs and MAPs are rather controversial, and no conclusive biological functions have been attributed to them.

In fish, MASPs have been cloned and analyzed in amphioxus and common carp (*Cyprinus carpio*)^23^,^24^,^25^. In common carp, MASP2 was found to be involved in the catalytic activation of C4^25^. Except for this, the immune function of MASPs in fish is entirely unknown. No reports on fish MAP have been documented. In a previous study, we identified and characterized three MBLs (named CsBML1 to 3) in the teleost fish *Cynoglossus semilaevis*^26^. In the present study, we aimed to better understand the immunological role of fish MAPs and MASPs by elucidating the biological properties of one MAP molecule and one MASP molecule from *C. semilaevis*.

## Results

### Sequence characteristics of two MASP family proteins

**Figure 1:**
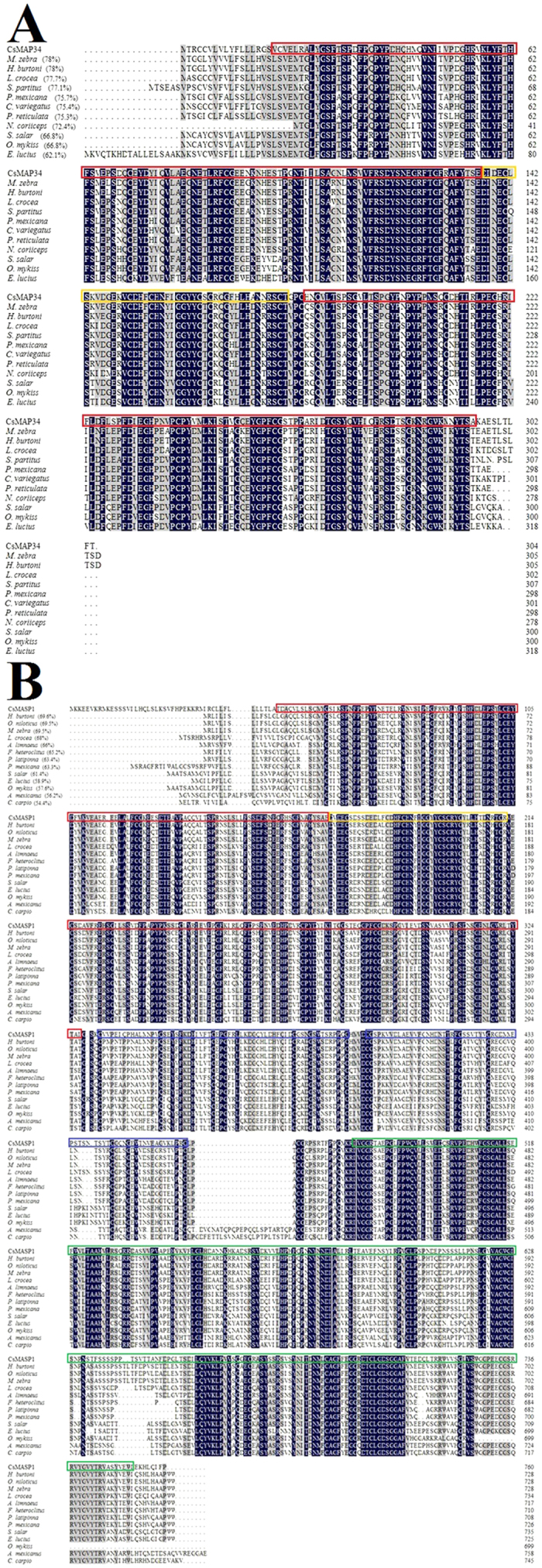
Alignment of the sequences of CsMAP34 (A) and CsMASP1 (B) homologues. Dots denote gaps introduced for maximum matching. Numbers in brackets indicate overall sequence identities between CsMAP34/CsMASP1 and the compared sequences. The consensus residues are in blue, the residues that are ≥75% identical among the aligned sequences are in grey. Red, yellow, blue, and green boxes indicate CUB domain, calcium-binding EGF-like domain, complement control protein (CCP) module, and trypsin-like serine protease domain, respectively. The GenBank accession numbers of the aligned sequences are as follows: (**A**) *Maylandia zebra* (zebra mbuna), XP_004554228.1; *Haplochromis burtoni* (African cichlid fish), XP_005915044.1; *Larimichthys crocea* (large yellow croaker), XP_010729754.1; *Stegastes partitus* (bicolor damselfish), XP_008280824.1; *Poecilia Mexicana* (Atlantic molly), XP_014846064.1; *Cyprinodon variegates* (sheepshead minnow), XP_015233610.1; *Poecilia reticulate* (guppy), XP_008411647.1; *Notothenia coriiceps* (Antarctic bullhead notothen), XP_010779504.1; *Salmo salar* (Atlantic salmon), XP_014001301.1; *Oncorhynchus mykiss* (rainbow trout), CDQ81624.1; *Esox lucius* (northern pike), XP_010890090.1. (**B**) *Haplochromis burtoni* (African cichlid fish), XP_014184888.1; *Oreochromis niloticus* (Nile tilapia), XP_005462796.1; *Maylandia zebra* (zebra mbuna), XP_014266317.1; *Larimichthys crocea* (large yellow croaker), XP_010748082.1; *Austrofundulus limnaeus* (annual killifish), XP_013886517.1; *Fundulus heteroclitus* (Atlantic killfish), XP_012712429.1; *Poecilia latipinna (Poecilia latipinna)*, XP_014894567.1; *Poecilia Mexicana* (Atlantic molly), XP_014836495.1; *Salmo salar* (Atlantic salmon), XP_014068886.1; *Esox lucius* (northern pike), XP_012988193.1; *Oncorhynchus mykiss* (rainbow trout), NP_001153950.1; *Astyanax mexicanus* (blind cavefish), XP_007233578.1; *Cyprinus carpio* (common carp), BAA86866.1.

The genome of *C. semilaevis* contains five sequences named MASP (GenBank accession numbers: XP_008316895.1, XP_008316896.1, XP_008307429.1, XP_008307430.1, and XP_008307432.1), of which, XP_008316896.1 and XP_008307430.1 were successfully cloned. Domain analysis showed that although named as MASP in the databank, XP_008316896.1 is in fact a homologue of MAP and therefore named CsMAP34 in this study. XP_008307430.1 is a MASP homologue and named CsMASP1. The deduced amino acid sequence of CsMAP34 has 304 residues, with a theoretical molecular mass of 34.3 kDa. CsMAP34 possesses two CUB domains (residues 18 to 136 and 183 to 295) and one calcium-binding EGF-like domain (residues 137 to 180) (Fig. S1A). The deduced amino acid sequence of CsMASP1 contains 760 residues, with a theoretical molecular mass of 84.9 kDa. CsMASP1 possesses two CUB domains (residues 47 to 168 and 215 to 327), one calcium-binding EGF-like domain (residues 169 to 212), two complement control protein (CCP) modules (residues 331 to 392 and 397 to 461), and one trypsin-like serine protease domain (residues 478 to 752) (Fig. S1B). CsMAP34 is homologous to the N-terminal segment (1 to 327 residues) of CsMASP1, with a sequence identity of 40.8%. CsMAP34 and CsMASP1 share 49.3% and 54.5% overall sequence identities with human MAP and MASP1, respectively. CsMAP34 and CsMASP1 share 45.1% and 39.5% overall sequence identities, respectively, with human MASP2. CsMAP34 and CsMASP1 share 62.1–78% and 54.4–69.6% overall sequence identities, respectively, with fish MASP (Fig. 1).

### Tissue expression patterns of CsMAP34 and CsMASP1 with and without bacterial infection

**Figure 2:**
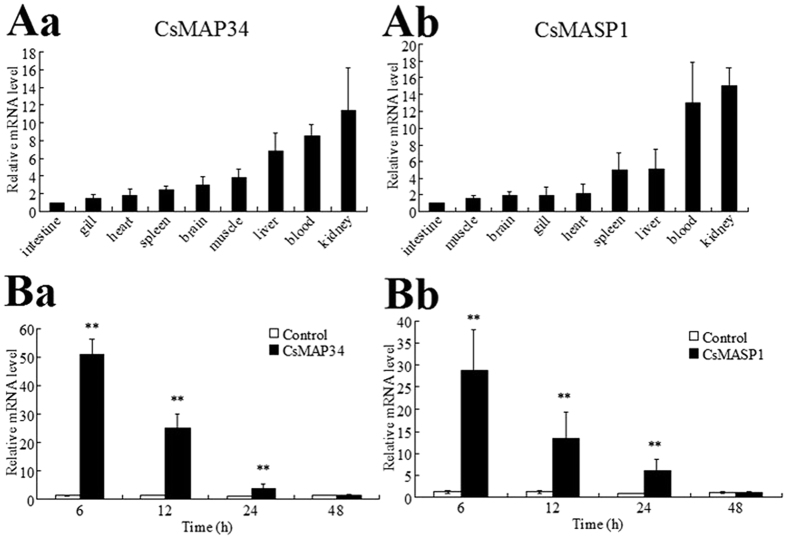
Expression of CsMAP34 and CsMASP1 in fish tissues under different conditions. (**A**) Under normal conditions, CsMAP34 (Aa) and CsMASP1 (Ab) expression in the intestine, gill, heart, spleen, brain, muscle, liver, blood, and kidney of tongues sole was determined by quantitative real time RT-PCR (qRT-PCR). For convenience of comparison, the expression levels in intestine were set as 1. (**B**) *C. semilaevis* were infected with or without (control) *Vibrio anguillarum*, and CsMAP34 (Ba) and CsMASP1 (Bb) expression in blood was determined by qRT-PCR at various time points. In all cases, data are the means of three independent experiments and presented as means ± SEM. ^*^P < 0.05, ^**^P < 0.01.

In the absence of bacterial infection, *CsMAP34* expression showed an increasing order in intestine, gill, heart, spleen, brain, muscle, liver, blood, and kidney, with the expression level in kidney being 11.5-fold higher than that in intestine (Fig. 2Aa). Likewise, *CsMASP1* expression showed an increasing order in intestine, muscle, brain, gill, heart, spleen, liver, blood, and kidney (Fig. 2Ab). When the fish were infected with *V. anguillarum*, a pathogenic bacterium to *C. semilaevis, CsMAP34* expression in blood was significantly upregulated at 6 h post-infection (hpi), 12 hpi, and 24 hpi (Fig. 2Ba). A similar expression pattern was observed with *CsMASP1* in blood (Fig. 2Bb).

### Capacity of rCsMAP34 and rCsMASP1 to interact with MBLs and bacteria

**Figure 3:**
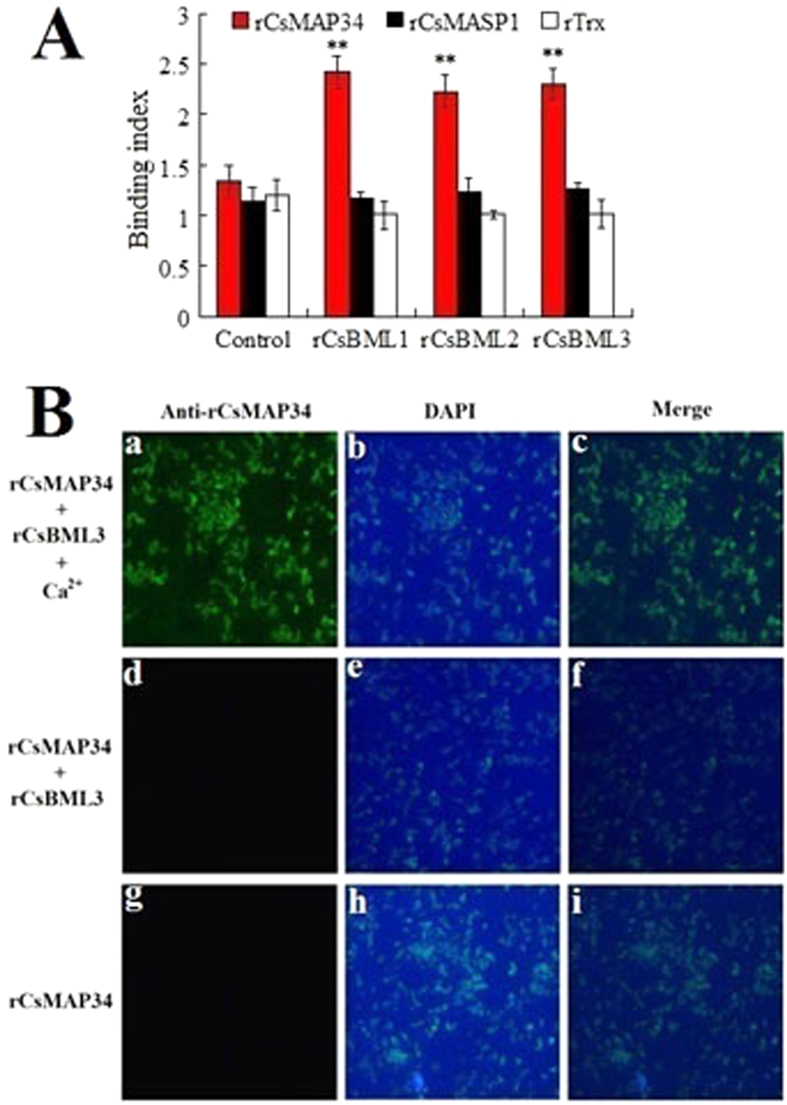
Binding of rCsMAP34 and rCsMASP1 to MBLs (A) and bacteria (B). (**A**) rCsMAP34, rCsMASP1, and rTrx were incubated with rCsBML1, rCsBML2, or rCsBML3 in the presence of *Vibrio anguillarum* and calcium. For control, rCsMAP34, rCsMASP1, and rTrx were incubated with HBSS in the presence of *Vibrio anguillarum* and calcium. Protein-protein binding was determined by ELISA. Data are the means of three independent experiments and presented as means ± SEM. ^**^P < 0.01. (**B**) rCsMAP34 was incubated with *V. anguillarum* alone (Bg and Bh) or with *V. anguillarum* plus rCsBML3 in the presence (Ba and Bb) or absence (Bd and Be) of calcium, and bacteria-associated rCsMAP34 was detected by FITC-labeled antibody and stained with DAPI. Bc, a merged image of Ba and Bb; Bf, a merged image of Bd and Be; Bi, a merged image of Bg and Bh.

To examine the binding potential of CsMAP34 and CsMASP1 to MBLs, purified His-tagged recombinant rCsMAP34 and rCsMASP1 (Fig. S2) were incubated with three *C. semilaevis* MBLs, i.e. rCsBML1, rCsBML2, and rCsBML3, in the presence or absence of *V. anguillarum* and calcium. The protein-protein binding was determined by ELISA, and the results showed that rCsMAP34 bound to all three MBLs only in the presence of *V. anguillarum* plus calcium, while no apparent interaction between rCsMAP34 and the MBLs was detected in the absence of bacteria or calcium (Fig. 3A and data not shown). In contrast, no apparent binding between rCsMASP1 and MBLs was detected either in the presence or absence of *V. anguillarum* (Fig. 3A and data not shown). Similar negative binding was also observed with the control protein rTrx (recombinant thioredoxin), which was purified under the same condition as rCsMAP34 and rCsMASP1. Fluorescence microscopy showed that rCsMAP34 was detected on *V. anguillarum* only in the presence of MBL (rCsBML3) and calcium, and that when rCsMAP34 was incubated alone with *V. anguillarum* or with *V. anguillarum* plus MBL but without calcium, no bacteria-associated rCsMAP34 was detected (Fig. 3B).

### Recruitment of rCsMASP1 by rCsMAP34

Since, as shown above, rCsMASP1 was unable to interact with MBLs, we wondered whether an indirect interaction between rCsMASP1 and MBL may occur through rCsMAP34. To examine this possibility, rCsMASP1 was incubated with rCsBML3 plus *V. anguillarum* and calcium in the presence of rCsMAP34 or rTrx. Subsequent ELISA analysis showed that in the presence of rCsMAP34, but not in the presence of rTrx, rCsMASP1 bound apparently to rCsBML3, with a binding index of 2.59 ± 0.2.

### Involvement of CsMAP34 in complement activation

#### Effect of CsMAP34 antibody on complement activation

**Figure 4:**
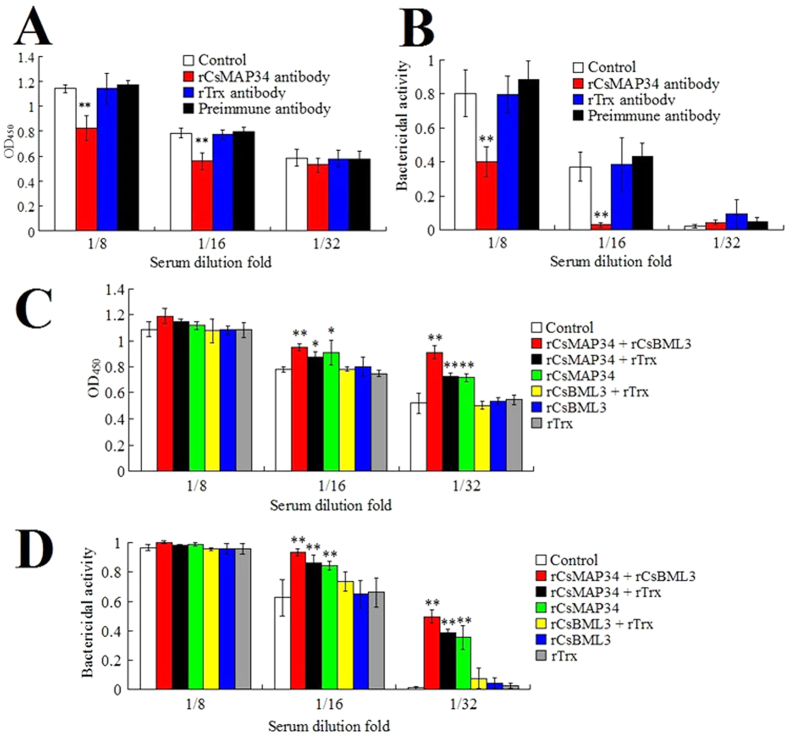
Effect of rCsMAP34 antibody and rCsMAP34 on complement activation. (**A** and **B**) *Cynoglossus semilaevis* serum in various dilutions was incubated with or without (control) rCsMAP34 antibody, rTrx antibody, or preimmune antibody. At 1 h after incubation, the hemolytic (**A**) and bactericidal (**B**) activities of the serum were determined. (**C** and **D**) *C. semilaevis* serum in various dilutions was incubated with or without (control) rCsMAP34 plus activated rCsBML3, rCsMAP34 plus rTrx, rCsMAP34 alone, activated rCsBML3 plus rTrx, activated rCsBML3 alone, or rTrx. At 1 h after incubation, the hemolytic (**C**) and bactericidal (**D**) activities of the serum were determined. Data are the means of three independent assays and presented as means ± SEM. ^*^P < 0.05; ^**^P < 0.01.

As shown in Fig. S3, ELISA detected production of CsMAP34 in the blood of *C. semilaevis*, especially after pathogen (*V. anguillarum*) challenge, which significantly increased the blood level of CsMAP34 in a time-dependent manner. With this observation, we examined whether CsMAP34 was required for complement activation. For this purpose, dilutions of *C. semilaevis* serum were incubated with rCsMAP34 antibody to block serum CsMAP34, and activation of the complement system was subsequently determined. The results showed that in the presence of anti-CsMAP34 antibody, the hemolytic activities of the serum at 8- and 16-fold dilutions were significantly reduced, whereas in the presence of rTrx antibody or preimmune antibody, no apparent effect was observed (Fig. 4A). Likewise, the bactericidal activities of the serum at 8- and 16-fold dilutions were significantly decreased by CsMAP34 antibody but not by rTrx antibody or preimmune antibody (Fig. 4B).

#### Effect of rCsMAP34 on complement activation

To investigate whether rCsMAP34 could affect complement activity, dilutions of *C. semilaevis* serum were incubated with rCsMAP34 alone, rCsMAP34 plus activated rCsBML3, or rCsMAP34 plus rTrx. Subsequent analysis showed that at 16- and 32-fold dilutions, the hemolytic activities of the serum treated with rCsMAP34 + rCsBML3, rCsMAP34 + rTrx, and rCsMAP34 alone were significantly increased compared to that of the untreated control serum, whereas the hemolytic activities of the serum treated with rCsBML3 + rTrx, rCsBML3, or rTrx were comparable to that of the control (Fig. 4C). Similarly, at 16- and 32-fold dilutions, the bactericidal activities of the serum treated with rCsMAP34 + rCsBML3, rCsMAP34 + rTrx, and rCsMAP34 were significantly higher than that of the control (Fig. 4D).

### Involvement of CsMASP1 in complement activation

**Figure 5:**
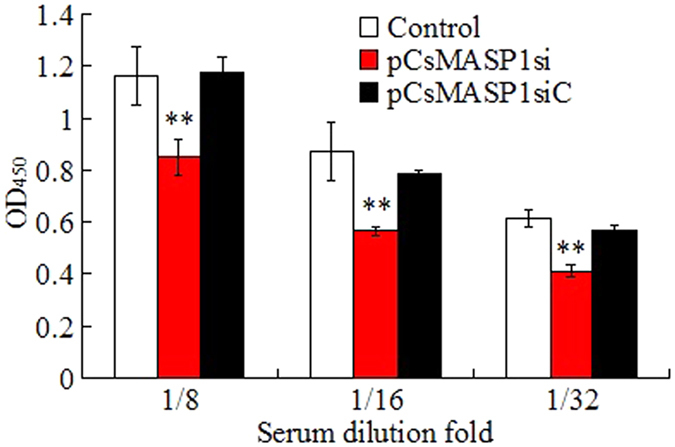
Effect of CsMASP1 knockdown on complement activation. Serum from *Cynoglossus semilaevis* treated with pCsMASP1si, pCsMASP1siC, or PBS (control) was serially diluted and determined for hemolytic activity. Data are the means of three independent assays and presented as means ± SEM. ^**^P < 0.01.

To examine the potential role of CsMASP1 related to complement activation, CsMASP1 expression in *C. semilaevis* was knocked down by RNA interference (RNAi). qRT-PCR analysis showed that in *C. semilaevis* administered with pCsMASP1si, which expresses a CsMASP1-targeting small interfering RNA (siRNA), the expression of CsMASP1 in blood, kidney, and spleen was significantly inhibited compared to that in the control fish (Fig. S4). In contrast, CsMASP1 expression in *C. semilaevis* administered with the plasmid pCsMASP1siC, which expresses a nonspecific siRNA, was comparable to that in the control fish (Fig. S4). After incubation with rabbit erythrocytes, the serum of the fish treated with pCsMASP1si exhibited significantly decreased hemolytic activities at 8x-, 16x- and 32x-dilution, whereas the serum of the fish treated with pCsMASP1siC exhibited hemolytic activities similar to that of the serum from the control fish (Fig. 5).

### Effect of rCsMAP34 on peripheral blood leukocytes (PBL) activity

**Figure 6:**
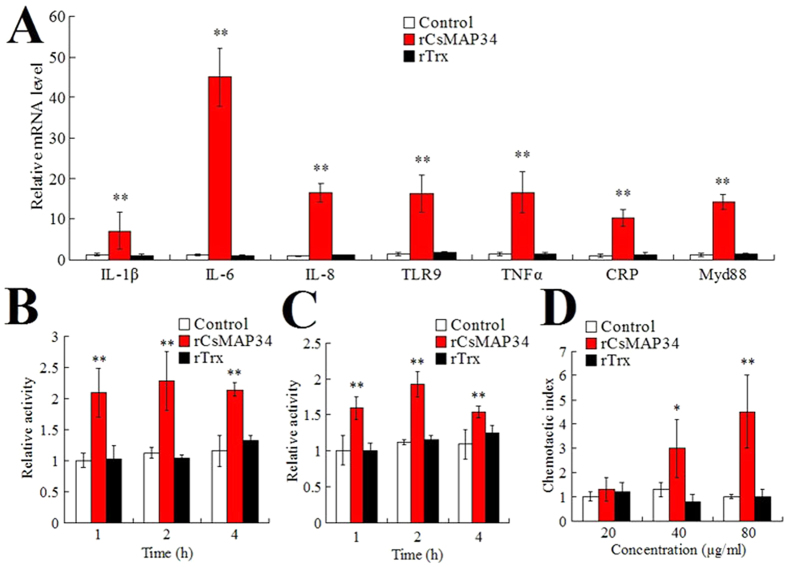
Activation of peripheral blood leukocytes (PBL) by rCsMAP34. (**A**) *Cynoglossus semilaevis* PBL were incubated with or without (control) rCsMAP34 or rTrx for 1 h, and immune gene expression was determined by quantitative real time RT-PCR. (**B** and **C**) *C. semilaevis* PBL were incubated as above for various hours, and the cells were then determined for respiratory burst (**B**) and acid phosphatase activity (**C**). (**D**) The chemotactic activity of rCsMAP34 or rTrx in various concentrations against PBL was determined using transwell migration assay. Data are the means of three independent assays and presented as means ± SEM. ^*^P < 0.05; ^**^P < 0.01.

Immunofluorescence microscopy showed that following incubation with *C. semilaevis* PBL, rCsMAP34 was observed on the surface of the cells, whereas rTrx similarly incubated with PBL failed to be detected on the cells (Fig. S5), suggesting a capacity of rCsMAP34 to bind to PBL. To examine whether this binding of rCsMAP34 had any impact on the activity of PBL, the cells were subjected to analysis of immune gene expression, respiratory burst, acid phosphatase activity, and chemotactic activity. The results indicated that, of the immune genes examined, i.e. interleukin (IL)-1β, IL-6, IL-8, tumor necrosis factor (TNF) α, toll-like receptor (TLR) 9, C-reactive protein (CRP), and myeloid differentiation factor (Myd) 88, all were significantly enhanced in expression by treatment with rCsMAP34 but not by treatment with rTrx (Fig. 6A). At 1 h, 2 h, and 4 h of incubation with rCsMAP34, but not with rTrx, the respiratory burst and acid phosphatase activities of PBL were significantly increased (Fig. 6B and C). Transwell migration assay showed that rCsMAP34, but not rTrx, could induce migration of PBL in a dose dependent manner (Fig. 6D). However, when rCsMAP34 was present at both the upper and lower chambers of the transwell, no apparent PBL migration was observed (data not shown), suggesting that PBL migration was induced by the presence of rCsMAP34.

## Discussion

In this study, we examined the expression and biological effects of *C. semilaevis* MAP and MASP. In mammals, the mRNAs of MASP1, MASP2, and MASP3 were detected in a broad range of tissues, in particular liver, intestine, and blood^27^, and MAP mRNAs were detected primarily in liver^28^,^29^. A previous study using a human monocyte cell line showed that the mRNA levels of MASP1 and MASP2 were increased and unaffected, respectively, by LPS and PMA^27^. In teleost fish, a MASP of zebrafish was induced in transcription by bacteria, whereas a MASP of common carp was downregulated in expression by parasite infection^30^,^31^, suggesting that MASP expression may differ in different fish and/or respond differently to different pathogens. Similar to the observation in zebrafish, we found that bacterial challenge enhanced the expression of CsMAP34 and CsMASP1. In the absence of bacterial infection, CsMAP34 and CsMASP1 expression occurred in different tissues with varying levels. Since the fish were clinically healthy, this difference in tissue expression was not likely due to health problems. Compared to other tissues, blood and kidney, which are the major organs of immunity, exhibited the highest levels of CsMAP34 and CsMASP1 expression, suggesting a participation of CsMAP34 and CsMASP1 in the immune surveillance of *C. semilaevis*. Following *V. anguillarum* challenge, the blood level of CsMAP34 protein was significantly increased. These results are in agreement with the structure-based prediction that CsMAP34 and CsMASP1 were MAP and MASP homologues, which in mammals are known to be part of the MBL pathway activation complex^32^,^33^.

In the lectin pathway of complement activation, MBLs act as recognition molecules, while MASPs are key enzymes for activating the cascade^19^. MBL binds to patterns of neutral sugars, for example N-acetyl-D-glucosamine and mannose, presented on the surfaces of various pathogens^34^,^35^. Binding of MBL to a target pathogen results in activation of the MASPs and subsequent activation of the lectin pathway^3^. Previous reports indicated that MASP2 could autonomously initiate the complement cascade by binding MBL^4^,^36^. However, other reports showed that addition of MBL allowed the formation of MASP1 and MASP2 complex, as well as other combinations of MASPs and MAPs^8^,^37^. Meanwhile, reports also showed that MASP1 activated MASP2, thereby activating the lectin pathway^2^,^17^,^18^. Hence, the functions of MASPs and MAPs appear to be rather complex. In *C. semilaevis*, our previous study demonstrated that recombinant CsBMLs were able to bind and agglutinate *V. anguillarum* in the presence of calcium^26^. In the present study, we found that rCsMAP34 could not bind to rCsBMLs alone but could bind to rCsBMLs activated by *V. anguillarum*, suggesting that bacteria-associated rCsBMLs may differ from free rCsBMLs in certain structural features required for rCsMAP34 recognition/interaction. In contrast to rCsMAP34, rCsMASP1 bound to rCsBMLs not by itself but only in the presence of rCsMAP34. These results suggest that CsMAP34 recruits CsMASP1 to bacteria-associated CsBMLs, and thus may control CsMASP1 activation.

In humans, MBL recognizes target molecules and induces activation of MASPs^38^, while MAP44 plays a regulatory role in controlling the lectin pathway^20^,^39^,^40^. One opinion is that MAP44 competes with MASP2 for binding to MBL, resulting in inhibition of complement activation^8^. MAP44 may inhibit complement activation, not simply by displacement of MASP2 from MBL, but also by displacement of either MASP1 or MASP2, thus disrupting such co-complexes and inhibiting complement activation^37^. However, in our study, we found that the presence of rCsMAP34 significantly stimulated the hemolytic and bactericidal activities of *C. semilaevis* serum. Consistently, the presence of anti-rCsMAP34 antibody inhibited serum complement activity, which is most likely due to antibody blocking of the natural CsMAP34 in the serum. Furthermore, we observed that *in vivo* interference of CsMASP1 expression in *C. semilaevis* successfully reduced the hemolytic activity of serum complement. These findings, together with the recruitment effect observed above with rCsMAP34 on rCsMASP1 and rCsBML, support the hypothesis that formation of the CsMAP34-CsBML-CsMASP1 complex may activate the protease activity of CsMASP1, which leads to further activation of the lectin pathway cascade. As such, CsMAP34, though lacking protease activity in itself, promotes complement activation indirectly.

In mammals, studies with endothelial cells showed that MASP1 induced IL-6 and IL-8 production, which led to chemotaxis of neutrophils^41^, and that MBL could bind endothelial cells but did not activate them^42^, neither did the MBL-MASP1 complex affect the activation of endothelial cells by MASP1^43^; MAP44 may play a role in endothelial functional regulation, but is potentially independent of lectin pathway activation^44^. In our study, we found that rCsMAP34 bound to and induced the migration of PBL, and that rCsMAP34 promoted the respiratory burst and acid phosphatase activities of PBL, suggesting that rCsMAP34 was able to activate PBL. Consistently, the presence of rCsMAP34 significantly upregulated the expression of immune genes in PBL. Together these results indicate a regulatory effect of CsMAP34 on the activity of immune cells.

**Figure 7:**
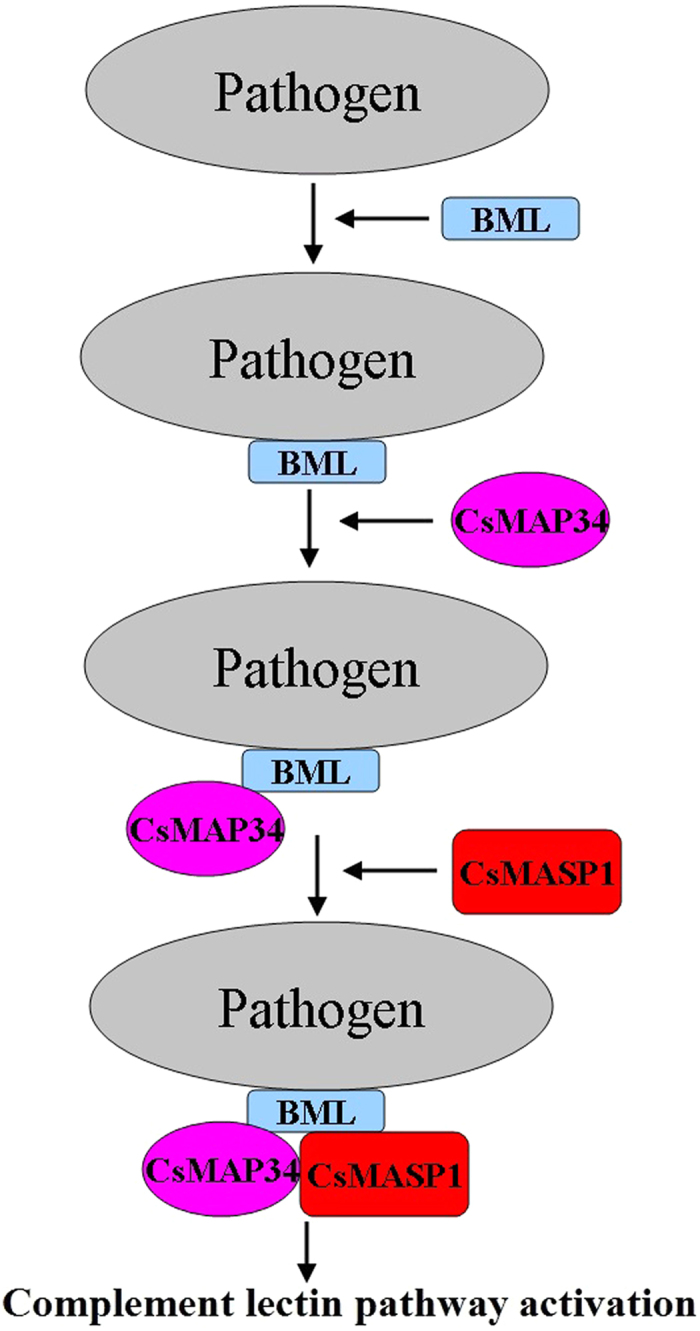
A proposed functional model of CsMAP34 and CsMASP1 in the lectin pathway activation of *Cynoglossus semilaevis*. Following pathogen infection, BML recognizes and binds the pathogen; CsMAP34 interacts with the pathogen-bound MBL and recruits CsMASP1; association with the CsMAP34-MBL complex activates CsMASP1 and leads to further activation of the lectin pathway cascade.

In conclusion, we demonstrate in this study that CsMAP34 and CsMASP1 are key factors involved in complement activation. We provided the first evidences that a teleost MAP, through its ability to bind to MASP1 and MBL, recruits MBL to MASP1, thus leading to MASP1 activation, which in turn likely stimulates the downstream activation process. Consequently, in contrast to the somewhat negative regulatory role of mammalian MAP, the teleost CsMAP34 exerts a positive effect on the activation of the lectin pathway. A working model of CsMAP34 and CsMASP1 was proposed based on these observations (Fig. 7). Furthermore, we observed for the first time that a teleost MAP participates in host immunity not only through the complement system but also through its regulatory effect on immune cells. These results add new insights into the biological function of MAP and MASP.

## Materials and Methods

### Ethics statement

All experiments involving live animals conducted in this study were approved by the Ethics Committee of Institute of Oceanology, Chinese Academy of Sciences. The methods were carried out in accordance with the relevant guidelines, including any relevant details.

### Fish

Half-smooth tongue sole (*C. semilaevis*) were purchased from a commercial fish farm in Shandong Province, China. Fish were maintained at 20 °C in aerated seawater and fed daily with commercial dry pellets. Before experiment, the fish were verified to be clinically healthy by plate count examination of bacterial recovery from the blood, liver, kidney, and spleen of randomly sampled fish, which confirmed that the fish were pathogen free. Plate count was performed as reported previously^45^. Briefly, liver, spleen and kidney were taken aseptically and homogenized in sterile PBS; blood was drawn from the caudal vein of the fish. The tissue homogenates and blood were plated in triplicate in Luria-Bertani (LB) agar plates, and the plates were incubated at 28 °C for 48 h. Before tissue collection, fish were euthanized by immersion in seawater containing 10 mg/L of tricaine methanesulfonate (Sigma, St. Louis, MO, USA) for 30 minutes.

### Bacterial strains and culture conditions

*Vibrio anguillarum* C312 is a bacterial pathogen isolated from diseased fish. *Escherichia coli* BL21(DE3) and DH5α were purchased from TransGen (Beijing, China). All strains were cultured in Luria-Bertani broth (LB) medium at 28 °C (for *V. anguillarum*) or 37 °C (for *E. coli*).

### Sequence analysis

The amino acid sequences of CsMAP34 and CsMASP1 (GenBank accession no. XP_008316896.1 and XP_008307430.1, respectively) were analyzed using the BLAST program at the National Center for Biotechnology Information (NCBI). Domain search was performed with the conserved domain search program of simple modular architecture research tool (SMART) version 4.0. Multiple sequence alignment was created with DNAMAN.

### Quantitative real time reverse transcription–PCR (qRT-PCR)

For qRT-PCR analysis of gene expression under normal physiological conditions, kidney, blood, intestine, gill, brain, muscle, heart, spleen, and liver were taken aseptically from five *C. semilaevis* (average 13.6 g) and used for total RNA extraction with EZNA Total RNA Kit (Omega Bio-tek, Doraville, GA, USA). One microgram of total RNA was used for cDNA synthesis with the Superscript II reverse transcriptase (Invitrogen, Carlsbad, USA). The sequence of CsMAP34 was amplified with primers CsMAP34F1 (5′-TACAGGCTTCCGAGCATTTTA-3′) and CsMAP34R1 (5′-TGGGTGGATAAGGGTTAGGGT-3′). The sequence of CsMASP1 was amplified with primers CsMASP1F1 (5′-TACTACTGCTCCTGTCGCTATGG-3′) and CsMASP1R1 (5′-TCAAACTGGAGGCGGATCTTA-3′). qRT-PCR was carried out in an Eppendorf Mastercycler (Eppendorf, Hamburg, Germany) using the SYBR ExScript qRT-PCR Kit (Takara, Dalian, China) as described previously^46^. PCR efficiency and correlation coefficient were determined based on the slopes of the standard curves generated using serial 5-fold dilutions of cDNA. The efficiency was calculated as follows: E (%) = (10^−1/slope^ − 1) × 100^47^. Melting curve analysis of amplification products was performed at the end of each PCR to confirm that only one PCR product was amplified and detected. The expression level of CsMAP34 and CsMASP1 was analyzed using comparative threshold cycle method (2^−ΔΔCT^) with β-actin (ACTB) as an internal reference^48^.

For qRT-PCR analysis of gene expression during bacterial infection, *V. anguillarum* was cultured at 28 °C to an OD_600_ of 0.8; cells were washed with phosphate-buffered saline (PBS) and resuspended in PBS to 2 × 10^6^ CFU/ml. *C. semilaevis* were divided randomly into two groups and injected intraperitoneally with 50 μl *V. anguillarum* or PBS. Blood was taken from the fish (five at each time point) at 6 h, 12 h, 24 h, and 48 h post-bacterial infection. Total RNA extraction, cDNA synthesis, and qRT-PCR were performed as described above. The experiment was performed three times, and the data are given in terms of relative mRNA, expressed as means plus or minus standard errors of the means (SEM).

### Plasmid construction

To construct pEtCsMAP34, which expresses recombinant CsMAP34 (rCsMAP34) with Trx and His tags, the coding sequence of CsMAP34 without signal peptide sequence was amplified by PCR with primers CsMAP34F2 (5′-GATATCATGGTGGAACTCAGGGCGTTATA-3′, underlined sequence, EcoRV site) and CsMAP34R2 (5′-CGATATCTGTAAAGAGAGTGAGGGACTCAG-3′, underlined sequence, EcoRV site). The PCR product was ligated with the T−A cloning vector T-Simple (TransGen Biotech., Beijing, China), and the recombinant plasmid was digested with EcoRV to retrieve the CsMAP34-containing fragment, which was inserted into pET32a (Novagen, San Diego, USA) at the EcoRV site, resulting in pEtCsMAP34. To construct pEtCsMASP1, which expresses Trx- and His-tagged recombinant CsMASP1 (rCsMASP1) containing the CUB-EGF-CUB-CCP-CCP domains (residues 38 to 461), PCR was conducted with primers CsMASP1F2 (5′-GATATCATGCTCTTCCTGCTTCTCCTGAC-3′, underlined sequence, EcoRV site) and CsMASP1R2 (5′-GATATCACAGCGGGGCAGTTTGACT-3′, underlined sequence, EcoRV site), and the PCR product was inserted into pET32a as above.

### Purification of recombinant proteins and preparation of antibody

*E. coli* BL21 (DE3) was transformed with pEtCsMAP34, pEtCsMASP1, and pET32a (which expresses the Trx tag). The transformants were cultured in LB medium at 37 °C to mid-log phase, and the expression of rCsMAP34, rCsMASP1, and rTrx was induced by adding isopropyl-β-D-thiogalactopyranoside to a final concentration of 1 mM. After growth at 16 °C for an additional 16 h, the cells were harvested by centrifugation, and recombinant proteins were purified using nickel-nitrilotriacetic acid columns (GE Healthcare, Piscataway, USA), as recommended by the manufacturer. The purified proteins were reconstituted as described previously^49^. The reconstituted proteins were treated with Triton X-114 to remove endotoxin as reported previously^50^. The proteins were dialyzed for 24 h against PBS and concentrated using PEG20000 (Solarbio, Beijing, China). The concentrations of the purified proteins were determined using the Bradford method with bovine serum albumin as a standard. Mouse antibodies against rCsMAP34, rCsMASP1, and rTrx were prepared as described previously^51^. The antibodies were purified using rProtein G Beads (Solarbio, Beijing, China). The specificity and titer of the serum antibodies were determined by Western immunoblot and enzyme-linked immunosorbent assay (ELISA) as reported previously^52^.

### Binding of rCsMAP34 and rCsMASP1 to MBLs

*V. anguillarum* was cultured as described above. The cells were washed with Hank’s Balanced Salt Solution (HBSS) (Solarbio, Beijing, China) containing no Mg^2+^ or Ca^2+^ and resuspended in HBSS to 10^8^ CFU/ml. rCsBML1, rCsBML2, and rCsBML3^26^ were mixed with the bacterial suspension to a final concentration of 80 μg/ml. CaCl_2_ was then added to the mixtures to the final concentration of 0.5 mM. The control sample was the bacterial suspension containing 0.5 mM CaCl_2_ but without any added protein. The mixtures were incubated at 22 °C for 1 h. Protein-protein interaction was determined by ELISA as reported previously^53^. Briefly, the mixtures were added to ELISA plates (100 μl/well), and the plates were incubated at 4 °C for overnight. After incubation, 5% skim milk powder in PBS was added to the plates (250 μl/well), and the plates were incubated at 22 °C for 1 h. The plates were washed with PBST (PBS containing 0.05% Tween-20) three times, and 80 μg/ml rCsMAP34, rCsMASP1, rTrx, or HBSS was added to the plates (100 μl/well). The plates were incubated at 22 °C for 2 h and washed as above. Mouse antibodies against rCsMAP34, rCsMASP1, and rTrx or preimmune antibody (1/1000 dilution) were added to the respective plates (100 μl/well). The plates were incubated at 37 °C for 1 h and washed three times in PBST. Goat anti-mouse IgG-horseradish peroxidase (HRP) antibody (Tiangen, Beijing, China) (1/1000 dilution) was added to the plates, and the plates were incubated at 37 °C for 1 h. After incubation, the plates were washed five times in PBST. Color development was performed using the TMB Kit (Tiangen, Beijing, China). The plates were read at 450 nm with a Precision microplate reader (Molecular Devices, Toronto, Canada). Positive readings were defined as at least twice of that of the control. The assay was performed three times, and the results are expressed as binding index, which is defined as follows: *A*_450_ of protein containing sample/*A*_450_ of control sample.

### Immunofluorescence microscopy

To examine protein binding to bacterial cells, *V. anguillarum* was cultured and prepared as above. rCsBML3 or HBSS (control) was mixed with the bacterial suspension to a final concentration of 80 μg/ml in the presence or absence of 0.5 mM CaCl_2_. Four hundred microliters of mixture was dropped onto a glass slide, and the slide was incubated at 22 °C for 4 h. After incubation, the slide was washed three times with PBS, and 400 μl of rCsMAP34 (80 μg/ml) was added to the slide in the presence of 0.5 mM CaCl_2_. The slide was incubated at 22 °C for 2 h and washed three times with PBS to remove nonspecific binding. Mouse anti-rCsMAP34 antibody (1/1000 dilution) was added to the slide. The slide was incubated at 37 °C for 2 h and washed as above. Fluorescein isothiocyanate (FITC)-labeled goat antimouse IgG (Bioss, Beijing, China) (1/1000 dilution) was added to the slide. The slide was incubated at 37 °C for 1 h in dark and then washed as above. Bacterial cells were stained with 4,6-diamino-2-phenyl indole (DAPI) (Invitrogen, USA) according to manufacturer’s instruction. The cells were observed with a fluorescence microscope (Nikon E800, Japan).

To examine protein binding to PBL, blood was collected from the caudal veins of *C. semilaevis*, and PBL were prepared with 61% Percoll and cultured in L-15 medium (Thermo Scientific HyClone, Beijing, China) as described previously^54^. PBL were resuspended in HBSS containing 0.5 mM CaCl_2_ to 10^7^ cells/ml. rCsMAP34 or rTrx was added to PBL suspension to a final concentration of 80 μg/ml. The cells were incubated at 22 °C for 2 h and centrifuged at 300 *g* for 10 min. The cells were collected, washed three times with HBSS, and resuspended in HBSS. Mouse monoclonal antibody against His tag (Bioss, Beijing, China) was added to the cells (1/1000 dilution), and the cells were incubated at 22 °C for 2 h. The cells were centrifuged, washed, and resuspended in HBSS as above. PBL-bound protein was detected as above, and the cells were observed with a fluorescence microscope as above.

### Binding of rCsMASP1 to MBLs via rCsMAP34

*V. anguillarum* suspensions (10^8^ CFU/ml) 80 μg/ml rCsBML3 plus 80 μg/ml rCsMAP34, rTrx, or HBSS were prepared. CaCl_2_ was added to each suspension to the final concentration of 0.5 mM. The mixtures were incubated at 22 °C for 1 h and placed into ELISA plates. After incubation at 4 °C for overnight, 5% skim milk powder in PBS was added to the plates, and the plates were incubated at 22 °C for 1 h. The plates were washed three times with PBST, and 100 μl of 80 μg/ml rCsMASP1 or HBSS was added to the plates. CaCl_2_ was added to the plates to the final concentration of 0.5 mM. The plates were incubated at 22 °C for 2 h and washed as above. Mouse anti-rCsMASP1 antibody or preimmune antibody (1/1000 dilution) was added to the plate. ELISA was performed as above.

### Serum hemolytic and bactericidal activity

To examine the effect of rCsMAP34 on serum hemolytic and bactericidal activity, *C. semilaevis* serum was diluted serially in HBSS. Serum dilutions containing rCsMAP34, rCsMAP34 + rCsBML3, rCsMAP34 + rTrx, rCsBML3 + rTrx, rCsBML3, and rTrx were prepared, in which all proteins were at the final concentration of 80 μg/ml. CaCl_2_ was added to each mixture to the final concentration of 0.5 mM. The control serum contained 0.5 mM CaCl_2_ but without any added protein. The mixtures were incubated at 22 °C for 1 h. For hemolysis assay, rabbit red blood cells (RRBC) (purchased from Guangzhou Future Technology Co., Ltd, Guangzhou, China) were washed and resuspended in HBSS. Ten microliters of RRBC suspension were added with 50 μl serum mixture in a 96-well culture plate. The plate was incubated at 22 °C for 30 min. After incubation, the supernatant was collected by centrifugation and determined for absorbance at 450 nm. For bactericidal assay, the serum mixture was either untreated or being heated at 56 °C for 30 min. *E. coli* DH5a was cultured in LB medium to an OD_600_ of 0.8. The cell was washed and resuspended to 2 × 10^6^ CFU/ml in HBSS. The bacterial suspension was combined with an equal volume of heated or unheated serum mixture in the presence of 0.5 mM CaCl_2_. After incubation at 22 °C for 1 h, the sample was serially diluted and plated in triplicate on LB agar plates. The plates were incubated at 28 °C for 24 h, and the colonies that appeared on the plates were enumerated. The genetic nature of the colonies was verified by PCR. The bactericidal activity was defined as {1 − (number of cells from unheated sample/number of cells from heated sample)} × 100%.

To examine the effect of CsMAP34 antibody on serum hemolytic and bactericidal activity, the serum was diluted serially in HBSS. rCsMAP34 antibody, rTrx antibody, and preimmune antibody (1/500 dilution) were mixed with serum dilutions in the presence of 0.5 mM CaCl_2_. The same volume of HBSS was mixed the control serum with containing 0.5 mM CaCl_2_. The mixture was incubated at 22 °C for 1 h. Hemolysis and bactericidal assay were performed as above.

### CsMASP1 knockdown by RNA interference (RNAi)

CsMASP1 knockdown was achieved through DNA vector-based small interfering RNA (siRNA) technology as reported previously^55^. Briefly, to select CsMASP1 specific siRNA, three different siRNA targeting CsMASP1 were inserted into the siRNA expression vector pRNAT-CMV3.1 (GenScript, Piscataway, USA) at BamHI/AlfII sites, resulting in plasmids pCsMASP1si-1, pCsMASP1si-2, and pCsMASP1si-3. In addition, the plasmid pCsMASP1siC, which expresses a scramble siRNA, was constructed in the same fashion. To examine the efficiency of the siRNA plasmids, five groups (N = 5) of *C. semilaevis* (average 32.8 g) were injected i.m. with each of the plasmids (30 μg/fish) or with PBS. At 7 d post-plasmid administration, expression of CsMASP1 in blood, kidney, and spleen was determined by qRT-PCR as described above. The plasmid with the strongest inhibitory effect on CsMASP1 expression was renamed pCsMASP1si. This screening experiment was performed three times. The siRNA sequences expressed by pCsMASP1si and pCsMASP1siC are 5′-TGCGTAGTACCTGAAATCC-3′ and 5′-ATGCCGAACTAGTATCCTG-3′ respectively. To examine the effect of NAi on complement activation, serum was taken aseptically from five *C. semilaevis* administered with pCsMASP1si, pCsMASP1siC, or PBS as above at 7 d post-plasmid administration. The serum was diluted serially in HBSS, and hemolysis assay was performed as above.

### Immune gene expression

PBL (10^6^ cells/ml) prepared above were treated with 80 μg/ml rCsMAP34, rTrx, or HBSS (control) in the presence of 0.5 mM CaCl_2_ for 1 h. Total RNA was prepared from the cells with RNAprep Kit (Tiangen, Beijing, China) and used for qRT-PCR analysis of the expression of immune genes as described above. The PCR primers of the immune genes have been reported previously^56^,^57^,^58^,^59^.

### Chemotaxis analysis

Chemotaxis was performed as reported previously^60^. Briefly, rCsMAP34 and rTrx were diluted in HBSS to 20 μg/ml, 40 μg/ml, and 80 μg/ml in the presence of 0.5 mM CaCl_2_. As a control, HBSS was similarly diluted. Each dilution (600 μl) was applied to the lower chamber of a 24-well Costar Transwell (Corning Costar Co., Cambridge, USA). The upper chamber containing a polycarbonate membrane with a 3-μm pore size was placed on the top of the lower chamber. PBL (~10^5^ cells) prepared above were added to the upper chamber, and the Transwell was incubated at 22 °C for 40 min. The number of cells that migrated into the lower chamber was counted using a microscope. The chemotactic index is presented as the fold-increase in the number of migrated cells induced by protein compared to that induced by HBSS.

### Respiratory burst and acid phosphatase activity assay

For respiratory burst assay, rCsMAP34, rTrx, or HBSS (control) was added to PBL in a 96-well tissue culture plate (~10^5^ cells/well) to a final concentration of 80 μg/ml. The plate was incubated at 22 °C for 1 h, 2 h, or 4 h. After incubation, the cells were determined for respiratory burst as reported previously^61^. For acid phosphatase activity assay, the above cells were lysed by adding 100 μl of 1% Triton X-100 to each well and incubation at 4 °C for 20 min. After incubation, acid phosphatase activity was determined using Acid Phosphatase Assay Kit (Beyotime, Beijing, China) according to manufacturer’s instruction.

### Statistical analysis

All experiments were performed three times. Statistical analyses were carried out with SPSS 17.0 software (SPSS Inc., Chicago, IL, USA). Data were analyzed with analysis of variance (ANOVA), and statistical significance was defined as *P* < 0.05.

## Additional Information

**How to cite this article**: Li, M.-f. *et al*. CsMAP34, a teleost MAP with dual role: A promoter of MASP-assisted complement activation and a regulator of immune cell activity. *Sci. Rep.* **6**, 39287; doi: 10.1038/srep39287 (2016).

**Publisher's note:** Springer Nature remains neutral with regard to jurisdictional claims in published maps and institutional affiliations.

## Supplementary Material

Supplementary Information
