# Supplementary material for: CsMAP34, a teleost MAP with dual role: A promoter of MASP-assisted complement activation and a regulator of immune cell activity
Source: Sci Rep. 2016 Dec 23;6:39287. doi: 10.1038/srep39287 (PMC5180248; doi:10.1038/srep39287)
Supplement: Supplementary Information [file srep39287-s1.doc]

**CsMAP34, a teleost MAP with dual role: A promoter of MASP-assisted complement activation and a regulator of immune cell activity**

Mo-fei Li, Jun Li, Li Sun

**Supplemental data**

**Figure S1．**Domain structures of CsMAP34 (A) and CsMASP1 (B). CUB, complement subcomponent C1r/C1s-like domain; EGF CA, calcium-binding EGF-like domain; CCP, complement control protein; Tryp_SPc, Trypsin-like serine protease.

**
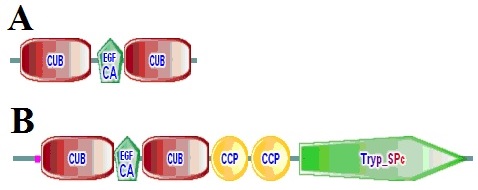
**

**Figure S2.** SDS-PAGE analysis of rCsMAP34, rCsMASP1, and rTrx. Unpurified rCsMAP34, rCsMASP1, and rTrx (lanes 2, 4, and 6 respectively) and purified rCsMAP34, rCsMASP1, and rTrx (lanes 3, 5, and 7 respectively) were analyzed by SDS-PAGE and viewed after staining with Coomassie brilliant blue R-250. Lane 1, protein markers.


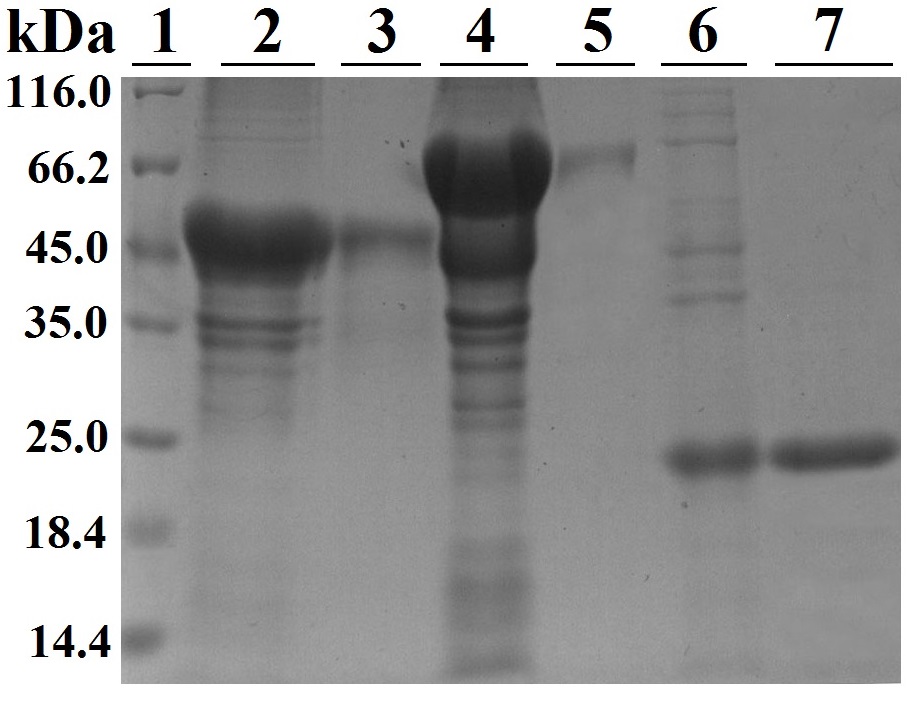


**Figure S3.** Production of CsMAP34 in *Cynoglossus semilaevis* blood. *C. semilaevis* were infected with *Vibrio anguillarum* or PBS (control), and CsMAP34 in serum was detected by ELISA at various hours post-infection. Data are the means of three independent experiments and presented as means ± SEM. ^**^*P* < 0.01.


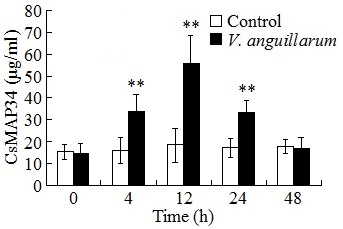


**Figure S4.** Expression of CsMASP1 in pCsMASP1si-administered fish. *Cynoglossus semilaevis* were administered with pCsMASP1si, pCsMASP1siC, or PBS (control), and *CsMASP1* expression in blood, kidney and spleen was determined by quantitative real time RT-PCR at 7 d post-plasmid administration. In each case, the expression level of the control fish was set as 1. Data are the means of three independent experiments and presented as means ± SEM. ^**^*P* < 0.01.


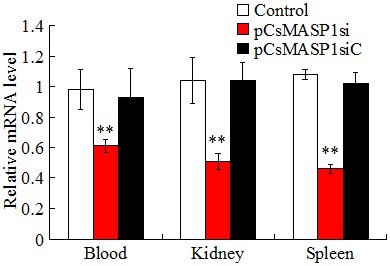


**Figure S5.** Binding of rCsMAP34 to peripheral blood leukocytes (PBL). PBL were incubated with rCsMAP34 (A and B) or rTrx (D and E), and cell-bound proteins were detected with FITC-labeled antibody. The cells were then examined under a fluorescence microscope with (A and D) or without (B and E) fluorescence light. C, a merged image of A and B; F, a merged image of D and E.

**
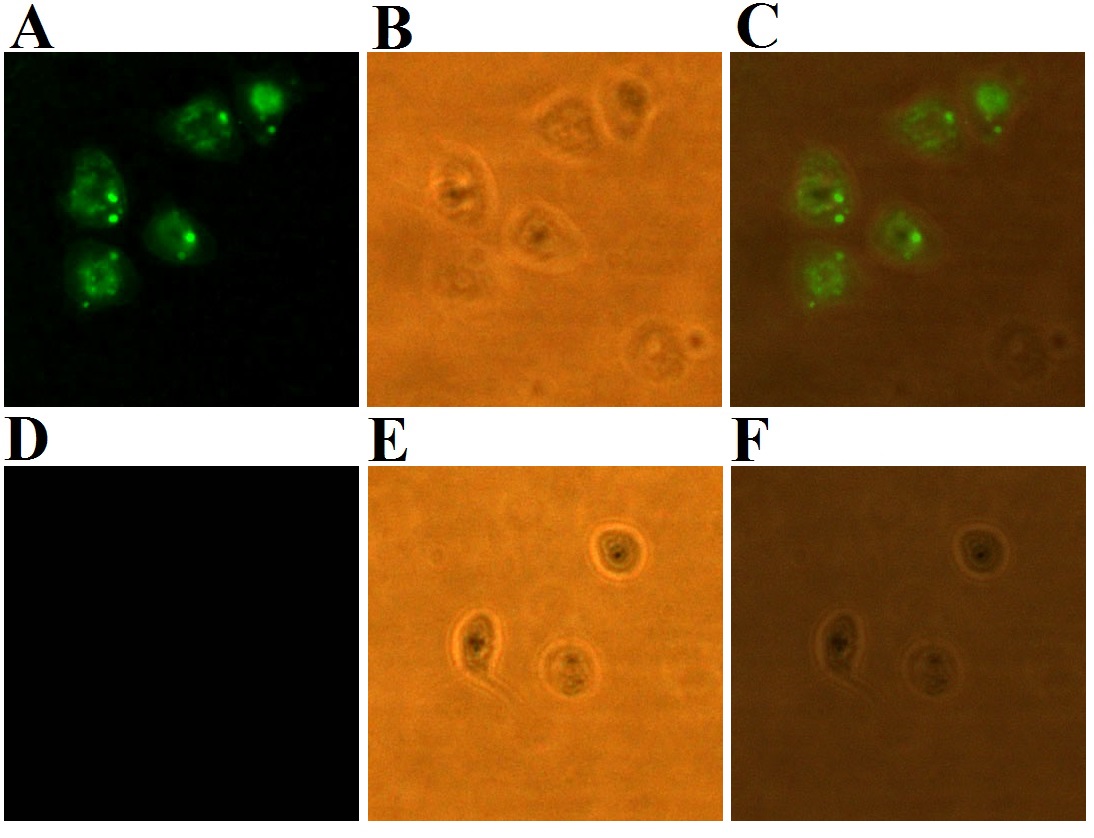
**
